# Conjunctival Pyogenic Granuloma Masquerading as Malignant Melanoma

**DOI:** 10.7759/cureus.17029

**Published:** 2021-08-09

**Authors:** Priyanka R Rao, Amjad U Furniturewala, Indumati Gopinathan, Akshay Gopinathan Nair

**Affiliations:** 1 Ophthalmology, Lokmanya Tilak Municipal Medical College and General Hospital, Mumbai, IND; 2 Cornea, Cataract and Refractive Surgery, Orbit Eye Hospital, Mumbai, IND; 3 Histopathology, Clinico-Path Labs, Mumbai, IND; 4 Ophthalmic Plastic Surgery & Ocular Oncology, Advanced Eye Hospital & Institute, Mumbai, IND

**Keywords:** melanoma, eyelid tumor, chalazion, eye cancer, pigmented tumor, conjunctiva

## Abstract

Pyogenic granuloma is a common, benign, vascular growth that often appears as a rapidly growing mass on mucus membrane-lined surfaces such as the conjunctiva. Conjunctival pyogenic granulomas are common following trauma, burst chalazion or ill-fitting prosthesis. Also known as 'lobular capillary hemangiomas', these lesions typically appear bright red, fleshy and pedunculated. Treatment options include excision, topical steroid therapy and topical beta-blocker therapy. In this communication, the authors describe a rapidly enlarging, pedunculated black coloured conjunctival mass lesion in a 44-year-old woman, who had a recent history of chalazia. Given the location and the clinical appearance, a melanocytic tumour was suspected and the mass was excised. Histopathology and immunohistochemical studies confirmed the diagnosis to be consistent with that of a a necrotic pyogenic granuloma. Pigmented lesions of the conjunctiva, especially rapidly enlarging ones, need to be viewed with a high degree of suspicion to rule out malignant melanoma. Rarely though, benign lesions such as pyogenic granulomas that undergo necrosis may masquerade as conjunctival melanomas.

## Introduction

Pyogenic granuloma is a benign, acquired, vascular lesion commonly seen on the conjunctiva. The term ‘pyogenic granuloma’ is a misnomer, as it typically is not associated with pus formation. Also known as ‘lobular capillary hemangiomas’ (LCHs), these lesions are commonly observed on the palpebral or bulbar conjunctiva following surgery, burst chalazion or even trivial trauma. Treatment options include excision, topical steroid therapy, intra-lesional steroid injection and topical beta-blocker therapy. Classically, conjunctival LCHs present as vascular, bright red, lobular masses [1]. In some cases, a tan colour may be seen due to intra-lesional fibrosis [2]. In this communication, we present the case of a deeply pigmented black conjunctival pyogenic granuloma masquerading as a melanoma.

## Case presentation

**Figure 1:**
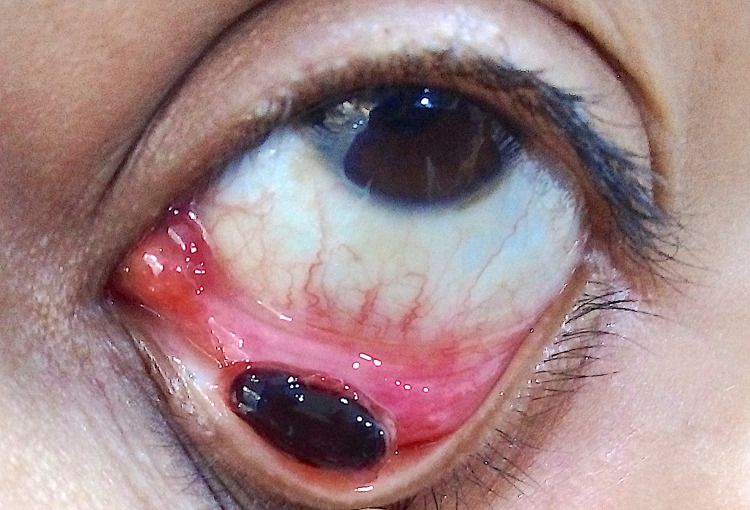
External clinical photograph External photograph of the left eye showing a smooth, glistening blackish mass arising from the conjunctival surface.

**Figure 2:**
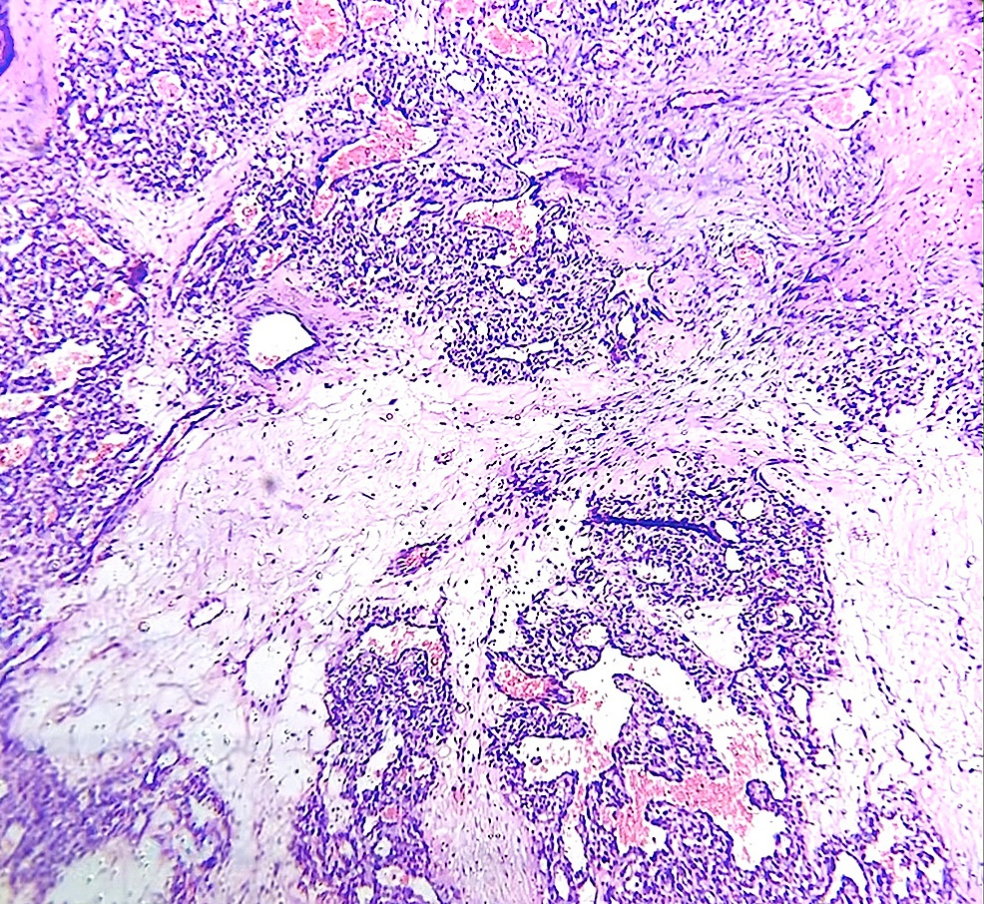
Histopathology image Microphotograph of the excised mass showing multiple vascular spaces with numerous engorged endothelial lined capillaries within the lobulated spaces (hematoxylin and eosin 40x).

A 44-year-old female presented with a pigmented dark mass lesion in the lower forniceal conjunctiva of the left eye. She had noticed it three days prior to presentation and had been referred to the ophthalmology clinic by her general practitioner to rule out conjunctival melanoma. On examination, the mass appeared smooth and had a glistening deeply pigmented surface with a small stalk attached to the conjunctival surface. Localised conjunctival hyperaemia was observed (Figure 1). There was no regional lymphadenopathy and visual acuity in both eyes was 20/20. Intraocular pressures and dilated fundus evaluation were unremarkable in both eyes. She gave a history of a lower lid chalazion, which had regressed spontaneously three weeks prior to this visit. The patient underwent an excision biopsy under local anaesthesia. Histopathological evaluation of the excised mass showed surface necrosis with multiple vascular spaces in the underlying stroma with numerous engorged endothelial lined capillaries within the lobulated spaces (Figure 2). Immunohistochemical staining for S100 and HMB 45 were negative. Based on the histological features, a diagnosis of LCH was made. At three months follow-up, no conjunctival scarring or recurrence was noted. 

## Discussion

The differential diagnoses for LCHs of the conjunctiva include suture granuloma, squamous papilloma, squamous-cell carcinoma and amelanotic melanoma [3]. In addition, conjunctival LCHs have been documented to masquerade as other conditions as well. AlHarkan and AlOdan reported the case of a four-year-old girl who underwent a strabismus surgery for exotropia and presented three weeks later with what appeared to be a large conjunctival abscess at the surgical site. The mass was excised, and histopathology showed inflammatory cells and histiocytes [4]. Atzrodt and colleagues reported the case of a 40-year-old male who had been diagnosed to have a stable conjunctival nevus [5]. Over subsequent visits, progression in the size of the lesion was documented and, eventually, the conjunctiva showed a vascularised, prominent, displaceable tumour with a prominent feeder vessel. On the clinical suspicion of progression of the nevus into an amelanotic conjunctival melanoma, excision was performed. Histopathological evaluation showed a conjunctival nevus combined with a pyogenic granuloma.

Charles and Kahn reported the case of a healthy 34-year-old white man who presented with a 10-day history of an enlarging brown mass of the left lower eyelid, along with mild discharge, bloody tears, oedema and erythema [2]. Examination showed a pedunculated, dark brown, mobile lesion arising from the tarsal conjunctiva. The mass was excised, and pathology showed vascular pattern of numerous blood-filled, capillary-sized channels surrounded by collars of fibrinoid necrosis. Pyogenic granuloma represents exuberant granulation tissue and is viewed as reactive hyperplasia of connective tissue in response to local irritants or trauma [6]. It is possible that as it increases in size, its blood supply ends up being inadequate; or twisting of the pedunculated mass on its pedicle could also lead to loss of blood supply - both mechanisms leading to subsequent infarction and necrosis [2]. The resultant surface necrosis blocks the reflection of red light from the tumour giving it a black appearance, which mimics a melanocytic lesion [2].

Typically a pyogenic granuloma of the conjunctival surface may be treated conservatively: topical steroid therapy or topical timolol therapy. However, in this case, it was the atypical clinical appearance and the recent history of increase in size that prompted a surgical intervention. Conjunctival melanomas arising from the nonbulbar (forniceal, palpebral and tarsal) conjunctiva are associated with a cumulative mortality rate of 43.6% (95% CI, 19.6%-77.9%) at 10 years of follow-up [7]. Therefore, pigmented conjunctival lesions must be addressed promptly. 

## Conclusions

Melanocytic lesions of the conjunctiva span across a wide spectrum of clinical conditions ranging from benign lesions such as conjunctival nevus or primary acquired melanosis (PAM) without atypia; to pre-malignant conditions such as PAM with atypia; as well as frankly malignant conditions such as conjunctival melanoma and pigmented variants of conjunctival squamous cell carcinoma. Therefore, pigmented lesions of the conjunctiva, especially rapidly enlarging ones, need to be viewed with a high degree of suspicion to rule out malignant melanoma. Rarely though, benign lesions such as pyogenic granulomas that undergo necrosis may masquerade as conjunctival melanomas.
